# Errors in Figure 2, Figure 3, and the Supplement

**DOI:** 10.1001/jamanetworkopen.2021.30836

**Published:** 2021-09-15

In the Original Investigation titled “Oral Anticoagulants for Nonvalvular Atrial Fibrillation in Patients With High Risk of Gastrointestinal Bleeding,”^1^ published August 16, 2021, there were errors in Figure 2, Figure 3, and the Supplement. In Figure 2, the *P* value for ischemic stroke in the comparison of rivaroxaban vs warfarin should have read .001. In Figure 3, the number of events in the reference group for major bleeding in the comparison of apixaban vs dabigatran should have read 599; the final comparison should have been dabigatran vs rivaroxaban; and the hazard ratio for ischemic stroke in the comparison of dabigatran vs rivaroxaban should have read 1.18. The Supplement has also been updated with new versions of eTable 2 and eFigure 2. This article has been corrected.^1^
